# Supplementary material for: Plasmon-Enhanced Blue-Light Emission of Stable Perovskite Quantum Dot Membranes
Source: Nanomaterials (Basel). 2019 May 19;9(5):770. doi: 10.3390/nano9050770 (PMC6566339; doi:10.3390/nano9050770)
Supplement: Supplementary file 1 [file nanomaterials-09-00770-s001.pdf]

Supporting Information

Plasmon-Enhanced Blue-Light Emission of Stable Perovskite Quantum Dot Membranes

Kai Gu [1], Hongshang Peng [1,2], Siwei Hua [1], Yusong Qu [1] and Di Yang [1,*]

* Correspondence: diyang@muc.edu.cn; Tel.: +86-1352-274-3208

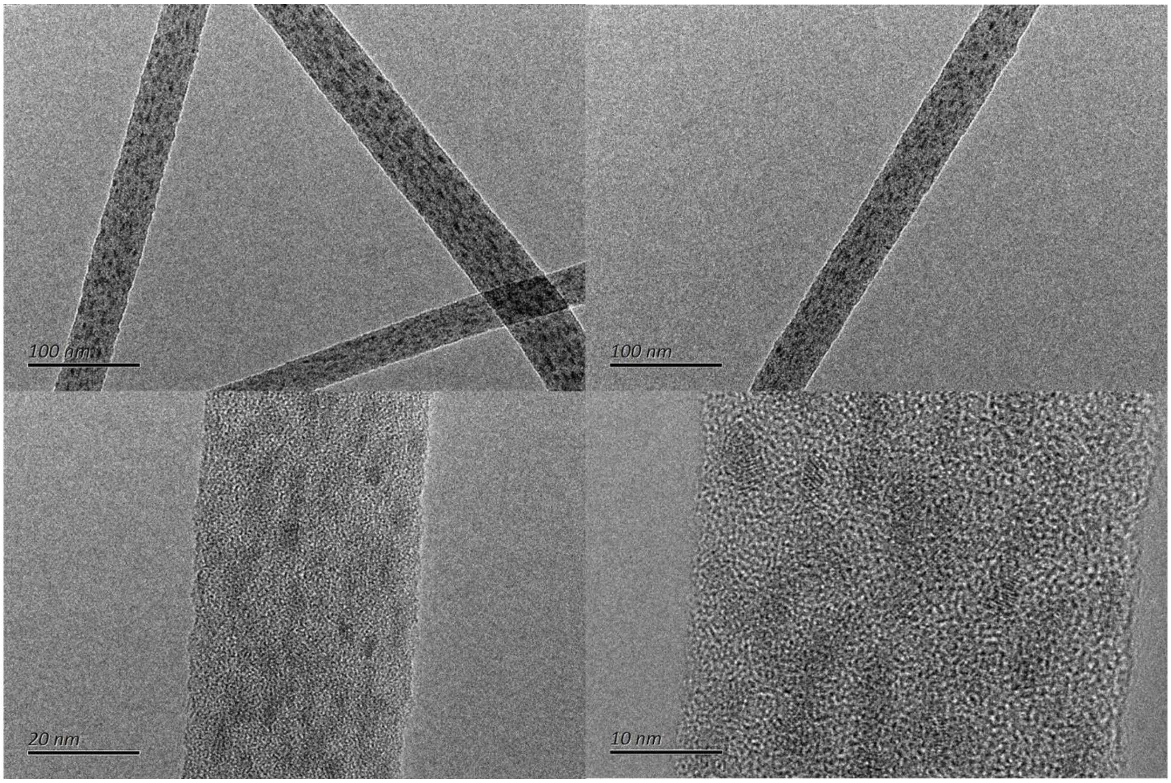

Figure S1. TEM and HRTEM images of PVDF-$MAPbBr_{1.2}Cl_{1.8}$ QDs nanofibers indicating that almost all PQDs are encapsulated inside the PVDF fibers.

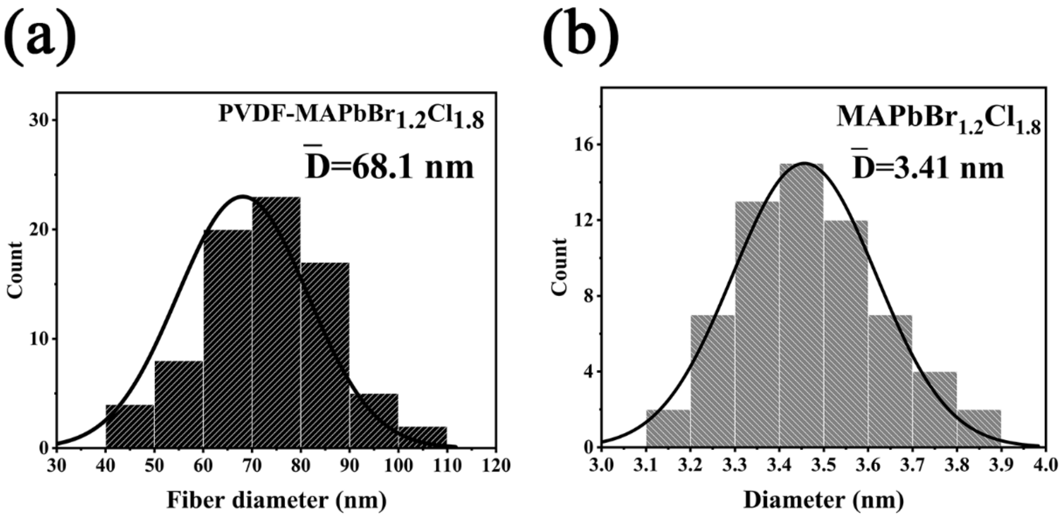

Figure S2. (**a**) The diameter statistics of the PVDF-$MAPbBr_{1.2}Cl_{1.8}$ fibers and (**b**) the size statistics of the $MAPbBr_{1.2}Cl_{1.8}$ QDs.

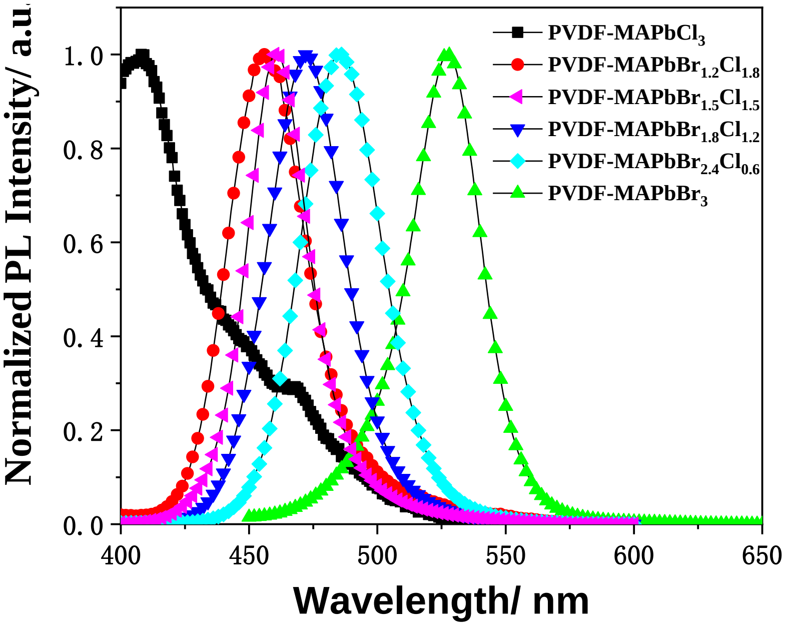

Figure S3. Normalized PL spectra of PVDF-$MAPbBr_{3-x}Cl_x$ with different the ratio of Cl^- and Br^-.

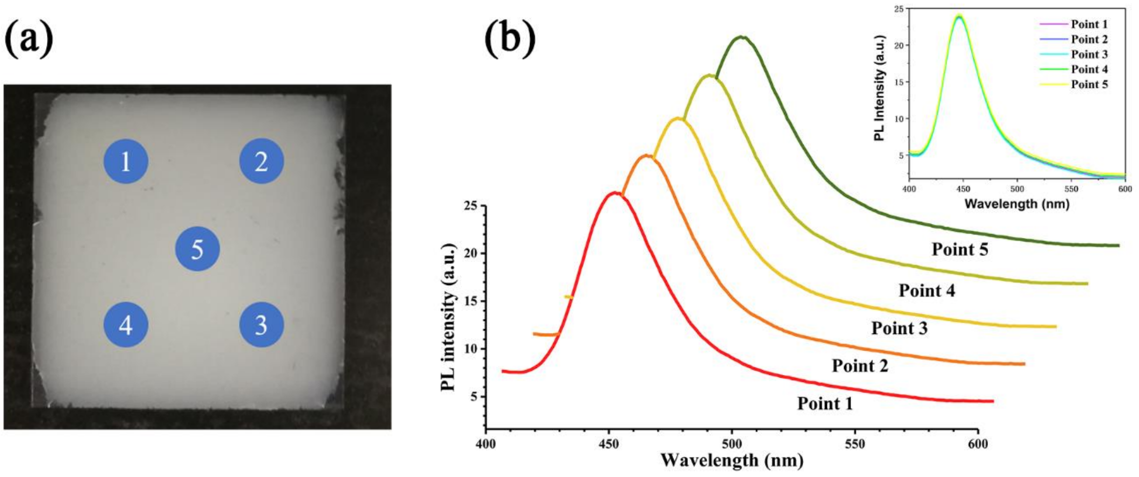

Figure S4. (**a**) Schematic diagram of five points on the PVDF-PQDs membrane; (**b**) The PL spectra of five points on the membrane. The inset shows overlap of the PL spectra of these five points.

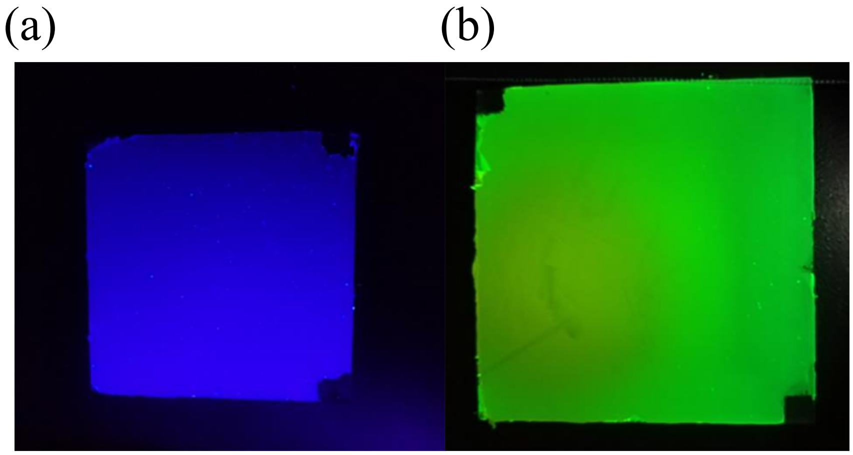

Figure S5. The optical images of (**a**) PVDF-$MAPbBr_{1.2}Cl_{1.8}$ and (**b**) PVDF-$MAPbBr_3$ films on FTO substrates under ultraviolet irradiation at ~365 nm wavelength.
